# Neural Differences between Covert and Overt Attention Studied using EEG with Simultaneous Remote Eye Tracking

**DOI:** 10.3389/fnhum.2016.00592

**Published:** 2016-11-23

**Authors:** Louisa V. Kulke, Janette Atkinson, Oliver Braddick

**Affiliations:** ^1^Department of Cognitive Developmental Psychology, Georg-Elias-Müller-Institute for Psychology, Georg-August University GöttingenGöttingen, Germany; ^2^Division of Psychology and Language Sciences, Faculty of Brain Sciences, University College LondonLondon, UK; ^3^Department of Experimental Psychology, University of OxfordOxford, UK

**Keywords:** attention, EEG, eye-tracking, fixation-shift paradigm, gap-overlap paradigm, non-verbal measures, attention shifts, covert attention

## Abstract

Research on neural mechanisms of attention has generally instructed subjects to direct attention covertly while maintaining a fixed gaze. This study combined simultaneous eye tracking and electroencephalogram (EEG) to measure neural attention responses during exogenous cueing in overt attention shifts (with saccadic eye movements to a target) and compared these with covert attention shifts (responding manually while maintaining central fixation). EEG analysis of the period preceding the saccade latency showed similar occipital response amplitudes for overt and covert shifts, although response latencies differed. However, a frontal positivity was greater during covert attention shifts, possibly reflecting saccade inhibition to maintain fixation. The results show that combined EEG and eye tracking can be successfully used to study natural overt shifts of attention (applicable to non-verbal infants) and that requiring inhibition of saccades can lead to additional frontal responses. Such data can be used to refine current neural models of attention that have been mainly based on covert shifts.

## Introduction

Attention is a set of cognitive processes that are fundamentally important for survival and development, allowing us to direct neurocognitive resources optimally for our behavioral goals. Attention enhances visual responses to stimuli, leading to an increased response of single cells towards attended stimuli in monkeys (e.g., Reynolds et al., 2000), and increased response amplitudes in human subjects (e.g., Eason et al., 1969; Keitel et al., 2013). Simultaneously, inhibitory mechanisms in the brain act to suppress responses to distracting stimuli (e.g., Kastner and Ungerleider, 2000). Visual response amplitudes decrease with increasing numbers of distracting stimuli, while attentional responses increase (e.g., Moran and Desimone, 1985; Keitel et al., 2013).

Most studies of the neural basis of attention have concentrated on the selection of stimuli in a particular region of visual space. The high resolution of the fovea means that this is typically accompanied by an overt shift of fixation toward the selected region. However, to identify selection processes within the brain, research paradigms have typically involved covert shifts of attention in which the participant attends to an extra-foveal region of space without a change of fixation (Harter et al., 1982; Posner et al., 1984; Rugg et al., 1987; Heinze et al., 1990, 1994; Luck et al., 1990; Mangun, 1995; Müller and Hillyard, 2000; Kelley et al., 2008; Tamber-Rosenau et al., 2011). It can be debated whether this form of attention represents a preparation for action, i.e., a saccade, which is not actually executed. On this view, covert and overt attention share processes, but covert attention additionally requires inhibition of the saccadic response.

The superior colliculus (SC) plays a crucial role in the generation of eye-movements, as it is directly connected to premotor circuits of the brain stem that generate saccades (e.g., Hanes and Wurtz, 2001; review of primate literature: Wurtz and Albano, 1980). Inactivation of SC neurons and lesions can lead to impairments in saccade execution (Sparks, 1988; Schiller and Tehovnik, 2005). Neggers et al. (2005) suggest that SC activation needs to reach a specific threshold for a saccade to be executed, making eye-movements more likely when SC activation in the relevant part of the spatial map is higher. As SC is a subcortical structure, its activation cannot directly be measured using electroencephalogram (EEG). However, SC is highly interconnected with cortical areas, in particular parietal areas and the frontal eye fields (FEF), which act as an integrated network in saccade initiation (Wurtz et al., 2001; Schall et al., 2011). Stimulation studies of V1 and V2 show that responses in the visual cortex can affect saccade execution (Schiller and Tehovnik, 2005). Furthermore human fMRI research suggests that the FEF can inhibit saccade neurons in the SC (Neggers et al., 2005), allowing control of the saccades that are elicited by SC. Thus EEG responses may be informative about the cortical components of this network.

Increased responses in visual areas have been suggested to occur through top-down modulation from the prefrontal cortex (PFC; for a review see Miller, 2000). Human (Neggers et al., 2005) and monkey (Hanes and Wurtz, 2001) research suggests that frontal regions, particularly FEF are also involved in saccade inhibition through the SC. FEF stimulation can produce saccades towards the contralateral side (Blanke et al., 1999) and FEF lesions impair the ability to shift gaze to the contralateral side and can lead to neglect (e.g., Crowne, 1983). Thus frontal responses may be important indicators of both covert and overt attention.

The EEG studies of attention in adults cited above instructed subjects to keep their gaze fixed while covertly shifting attention (e.g., Harter et al., 1982; Posner et al., 1984; Rugg et al., 1987; Heinze et al., 1990, 1994; Luck et al., 1990; Mangun, 1995; Müller and Hillyard, 2000), or to delay saccade execution after a cue until a “go-signal” was given (Eimer et al., 2007) since the eye movements in overt attention shifts can cause EEG artifacts (Corby and Kopell, 1972; Joyce et al., 2004). Studies of neuronal mechanisms of overt attention shifts using EEG are rare (e.g., Moster and Goldberg, 1991; Csibra et al., 1997). However it is important to study brain mechanisms of attention in non-verbal infants and other groups who may not comply with fixation instructions. In such cases, attention shifts must be studied based on eye movements, as in the Fixation Shift Paradigm (for a review see Atkinson and Braddick, 2012; e.g., Hood and Atkinson, 1993; Kulke et al., 2015b), which involves exogenous eye-movements without preceding cues. To achieve this, we tested “covert” attention shifts in a condition which required manual but not oculomotor responses to an exogenous stimulus. This differs from earlier studies of covert attention in the stimulus for an attention shift, but shares the requirement to avoid an overt shift of fixation.

Recent technological advances make it possible to study attention shifts using combined eye tracking and EEG (e.g., Kulke and Wattam-Bell, 2013; Dimigen, 2014; Kulke et al., 2015b; Meyberg et al., 2015), which gives us an opportunity to measure neural mechanisms of attention shifts without explicit verbal instructions. However, it is unclear how these tasks relate to previous paradigms with explicit instructions. The current study aimed to directly compare a manual response paradigm without an overt fixation shift, with a fixation-shift-paradigm in which saccades are directed to the target, recorded by remote eye tracking alongside simultaneous EEG recording. This simultaneous recording was used to allow gaze contingent control of the stimuli and to ensure that saccades were not included in the window of EEG analysis.

Healthy adults either overtly shifted their attention from a central stimulus to a peripheral target by making an eye movement towards it, or they covertly shifted their attention towards a peripheral target, making a manual response corresponding to the side of the target, while maintaining central fixation. To differentiate between visual and attentional components of the EEG, target stimuli randomly appeared on the left or right, or on both sides of the screen (giving the same visual input in both left and right hemisphere). While the different biomechanics will make saccadic and manual response times different, these times were expected to show a similar pattern between overt and covert attention shift tasks, with both saccadic and manual response latencies longer towards double than towards single stimuli since a decision component is required for double stimuli. The visual components of neural responses were expected to be similar for the identical visual stimuli in the overt and covert attention conditions. Previous studies have suggested that saccade planning can enhance brain activity related to visual processing (Saber et al., 2015) and that saccade planning may also involve fronto-central and prefrontal areas (Neggers et al., 2005). Therefore, we looked for differential frontal activation in overt compared to covert attention shift conditions.

## Materials and Methods

### Participants

Twenty-four students (20 female, 23 right-handed) from the UCL Psychology subject pool with a mean age of 21.3 years (SD = 2.4) participated in the study in return for monetary compensation (£10) or course credit. All had normal or corrected-to-normal vision and no known history of brain disease. One female subject was excluded because of a technical error. The number of recruited subjects was based on previous research examining attention shifts in adults (Heinze et al., 1990; Müller and Hillyard, 2000). The study was approved by the UCL ethics committee (Ref. number: CPB/2013/011 and CPB/2014/007) and written informed consent was obtained in accordance with the Declaration of Helsinki.

### Stimuli and Equipment

**Figure 1:**
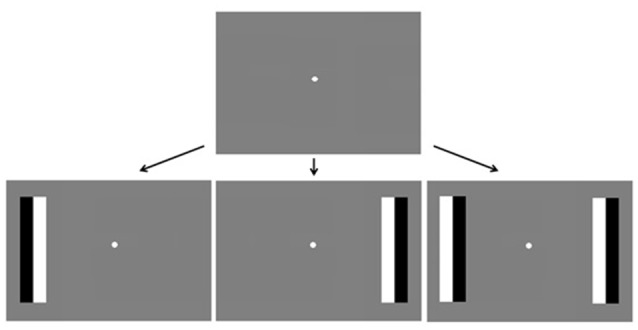
**Trial timing.** A fixation dot appears in the center of the screen followed by phase reversing bars on the left, right or both sides of the screen. Subjects respond by pressing buttons on a joypad (block 1) or by making eye-movements towards the bars (block 2).

A DELL computer with Linux operating system (Linux Mint 14), with MATLAB [version 7.11.0 (R2010b)] as the presentation program, was used to generate stimuli and present them on a 21.5” (54 cm) LCD monitor (Samsung) that extended over 35.8° × 22.8° of visual angle, running at a frame rate of 60 Hz. Stimuli were presented against a gray background with a mean luminance of 77 cd/m^2^. A joypad (Saitek USB V pad) was used to monitor participants’ manual responses. A remote eye-tracker, Tobii X120, was used to record the gaze-position of subjects during the experiment at a rate of 60 Hz. The average viewing distance was 65 cm, which approximates the distance at which the eye-tracker receives the best signal. This position was adjusted for each participant until the best possible eye-tracking signal was acquired. Stimulus sizes specified in visual angle are based on this distance. Stimuli were based on the Fixation Shift Paradigm (e.g., Hood and Atkinson, 1993; Kulke et al., 2015a) to facilitate comparison with previous research. At the beginning of each trial, a white dot subtending 0.7° visual angle appeared in the center of the screen for a randomized inter-trial interval between 0.5 s and 2.5 s. When the subject fixated on the dot, target stimuli randomly appeared on the left, right or on both sides of the screen at an eccentricity of 12.9°, while the dot remained present. The target stimuli were phase reversing black and white rectangular bars subtending 3.1° × 13.2°, with a reversal rate of 3 Hz (Figure 1) that appeared until the subject fixated on them for 330 ms (saccade condition) or until the subject made a manual response (manual response condition).

### Procedure

EEG was recorded (details below) while the subjects were engaged in the behavioral tasks. After a six-point eye-tracking calibration the main experiment started. In all conditions, a white fixation dot appeared in the middle of the screen. In the manual response condition, participants were instructed to fixate on this dot while keeping their gaze as still as possible and to press a button with their left or right thumb corresponding to the side on which the peripheral targets appeared. When targets appeared on both sides of the screen they were instructed to choose to press either the left or right button. In the saccade condition, participants were instructed to initially focus on the white central dot and to look at the target as soon as it appeared. When targets appeared on both sides of the screen they were instructed to choose either of the targets to fixate.

### Online Analysis of Eye-Tracking Data

During the experiment the eye-tracking data was accessed to monitor gaze positions. Whether a subject fixated on the initially presented central stimulus was determined by calculating the dispersion of measured gaze position from the center of the fixated object at the end of the random inter-trial interval. When this dispersion was less than 2.6° of visual angle for at least 20 samples (~330 ms), a target stimulus appeared in the periphery. If the subject looked at a peripheral stimulus, defined as the measured gaze position being in the area of 8° × 8° around the target stimulus for more than 20 samples (~330 ms), the stimulus automatically disappeared and the next trial began.

### Design

In a 2 × 2 × 2 within-subject design, the effects on behavioral response latency of the factor response type (manual or saccadic), number of targets (1 or 2) and screen side responded (left or right) were calculated. For the extracted neural responses, the effects on event-related potential (ERP) latencies and amplitudes of response type (manual or saccadic), number of targets (1 or 2), brain hemisphere (ipsilateral or contralateral to the target responded to) and brain side (left or right) were computed. Note that there are two measures for brain lateralization: *brain hemisphere* describes the lateralization in relation to the target (hemisphere ipsi- or contralateral to the stimulus that was reacted to) and *brain side* (left vs. right, irrespective of whether the target is on the left or right) because neural models of attention suggest a right-lateralized attention network (Corbetta et al., 1998; Corbetta and Shulman, 2011; Ptak, 2012). Mixed models were used for all multivariate analyses, including the main effect of subject ID as a random factor and full factorial models of all other factors as fixed factors, including main effects and interactions. Manual and saccadic conditions were tested in separate blocks with the manual blocks completed first. This fixed order was used since subjects could find it more difficult to inhibit saccades if they had been trained to look at the peripheral bars during the previous block. Other variables were randomly varied within these blocks. Both the manual and the eye-tracking conditions involved four blocks of 100 randomized trials. Subjects were presented with 200 single target trials (100 left, 100 right) and 200 double target trials in each of the manual and the saccadic response conditions. Short breaks were given between each block, with longer breaks occurring after 300 and 600 trials while the experimenter checked the electrode impedances and adjusted when necessary. The entire experiment lasted for approximately one and a half hours.

### Post Experiment Analysis

After completing the experiment, the eye-tracking data was processed for all samples and analyzed by a Matlab program for each trial.

If eye-position data was missing in a sample, the data in this sample was interpolated with the average of the previous sample and the first subsequent successful sample. If several successive samples were missing this procedure was repeated for all of them. The onset of a saccade was defined as the time point before a horizontal change of gaze-position on the screen by more than 2.2° of visual angle between two successive samples (i.e., a velocity higher than 132°/s).

Trials involving noisy eye tracking data were excluded according to the following criteria: (1) if the gaze position at the onset of the second stimulus was not on the screen (approximated as 19.6° × 11.0° from center of screen), indicating that the subject did not fixate the screen or that the eye tracker lost the signal; (2) if the trial contained too many large excursions in fixation position (>20% of samples differed by more than 2.2° of visual angle from the previous sample) indicating signal-loss from the eye-tracker; or (3) if the first saccade occurred earlier than 0.1 s after the appearance of the peripheral target, as it is very unlikely that those saccades were related to the appearance of the target and so were probably anticipatory saccades or unrelated to the stimulus (see Gómez et al., 1996).

Trials in which the first saccade occurred later than 5 s after target onset were registered as “sticky fixations” and excluded from the analysis of latencies (in line with the original infant research: Atkinson et al., 1992; Hood and Atkinson, 1993; Matsuzawa and Shimojo, 1997). Trials with the initial saccade to the wrong direction in single-target trials were registered as “misdirected saccades” and excluded from the analysis. Overall, 1.4% of trials (SD = 2.3%) were excluded. For overt attention shifts in valid correct trials, the latency (difference in time between target onset and onset of the first lateral saccade towards the side of the target) was calculated and averaged across the condition.

The eye-tracking data processing takes 30 ± 2 ms (Tobii Technology, 2010) and asynchrony between the eye tracking refresh pulse and the monitor’s refresh rate leads to an additional delay of up to 16.7 ms (at a rate of 60 Hz), on average 8.3 ms. The raw eye-tracking latencies were used for comparisons between conditions, and so a fairly constant delay should not affect the differences between them, but the absolute latencies may differ from the measured ones. This is only relevant for using the eye-tracking information to process EEG data, which was only done when excluding premature saccades to clean the EEG data. To avoid distortion of the EEG signal through saccades, a fixed correction of 50 ms was used, to ensure that no saccades were included in the time window for EEG analysis.

### EEG System

While the subjects were engaged in the behavioral tasks, their EEG activity was recorded at a rate of 250 Hz using Electrical Geodesics Inc. NetAmp300 amplifier and 128- channel Ag/AgCl electrode nets (Tucker, 1993). EEG was recorded on a separate computer (Macintosh) using Net Station 4.2 (© 1994–2006, Electrical Geodesics, Inc., Eugene, OR, USA). Electrode impedance was adjusted to less than 90 kΩ, with the majority of electrodes having an impedance of less than 40 kΩ (Ferree et al., 2001). This level was chosen to be comparable to parallel infant experiments, and has been shown to have a negligible effect on EEG data quality (Ferree et al., 2001; Richards, 2005).

### EEG Data Processing

**Figure 2:**
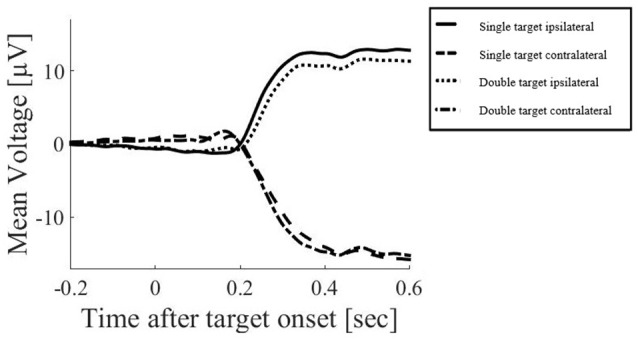
**Example of electroencephalogram (EEG) responses in the above mentioned electrode clusters around FC1 and FC2 over a long time window, averaged across all subjects.** Only the saccadic response condition is displayed here, demonstrating the major voltage deflections associated with the saccade (occurring at and after 200 ms in this example).

The timing of the EEG system was measured using a light-sensitive diode set up (for details on this method see Kulke, 2015) to ensure that triggers were aligned with visual events. Due to the physical properties of the EEG system, the amplifiers may introduce a delay, which was determined to be 24 ms and corrected for. An average reference was used. Eye-tracking data was used to exclude trials with early saccades (<180 ms after target onset after correction for potential delays), ensuring that EEG data was not confounded with eye movement artifacts. The EEG data analysis was programmed in MATLAB, using the following steps:

1. Butterworth filters were used for notch filtering around the line noise frequency [49–51 Hz], high-pass filtering (cut off: 0.01 Hz) and low-pass filtering (cut off: 25 Hz).
2. Segmentation of data into epochs of −200 ms to 180 ms around target onset. This window was selected to ensure that artifacts related to eye movements were excluded. Such artifacts include the electrical effects of the eye movement itself, but also the cerebral effects of the change in visual input as the saccade occurs and the visual display is swept across the retina. While current advancements in EEG data processing have suggested that eye-movement artifacts can be excluded using independent component analysis (ICA) or similar processing methods (for discussion see Plöchl et al., 2012), these methods do not necessarily exclude the visual effects which may have a common source with other visual responses recorded during the trial. To ensure that target-related responses were not confounded with such visual effects of the saccades, the current study focused on the response before eye-movements occur. Figure 2 illustrates an example of EEG responses over a long time window, demonstrating the major voltage deflections associated with the saccade (occurring at and after 200 ms in this example). The restriction of our analysis to the early time window does however limit our ability to compare results with studies that examined EEG responses occurring later after the initial stimulus for an attention shift (e.g., Harter et al., 1982; Heinze et al., 1990).
3. Noisy epochs and electrodes were then determined by using the median absolute deviation about the median (MAD; Hampel, 1974), as this is a measure that is fairly robust to noise (Hampel, 1974; Leys et al., 2013). Using a threshold of 3*MAD*1.483, values that showed higher deviations than this threshold were excluded from further analyses as “noisy trials”. Epochs were included if the following criteria were within this threshold calculated using the individual data in more than 70 channels: (a) SD over samples per trial; (b) range of amplitude between minimum and maximum amplitude value within epoch; (c) drift (difference in amplitude between the average period before and after target onset); (d) Maximum steps in data amplitude between successive samples; and if (e) the SD over samples per trial was bigger than 0.1 (i.e., the electrode received a signal).
4. Individual electrodes which did not meet these criteria within a specific epoch were interpolated using spherical spline interpolation, and only those that were acceptable according to (3) after interpolation were used for further analysis.
5. The average voltage during the baseline period ([−200; 0] ms before target onset) was used to correct the data from target onset onwards, individually for each trial and electrode. Scalp surface maps were created using spherical interpolation.

### ERP Measures

ERPs have commonly been quantified through peak amplitudes and latencies (e.g., Harter et al., 1982; Rugg et al., 1987; Mangun, 1995), and the same measures were taken in this study to maintain comparability with previous research.

Areas of interest were based on previous research. To extract occipital responses, an electrode cluster around electrodes O1 and O2 in the 10-10 system was extracted, which have been found to reliably show visual responses in previous studies (e.g., Mangun, 1995; Csibra et al., 1997; Mangun et al., 1997).

An early frontal negativity has previously been found to be influenced by spatial attention (e.g., Harter et al., 1982; Rugg et al., 1987; Heinze et al., 1990). It typically peaks in central and frontal sites, having higher amplitudes in the contralateral hemisphere (Rugg et al., 1987; Heinze et al., 1990). ERP responses from frontocentral areas were extracted in clusters around the electrodes FC3 and FC4 in the 10-10 system.

Different areas have been suggested to be involved in saccade control and execution, including frontal eye-fields (e.g., Guitton et al., 1985; Henik et al., 1994; Miller, 2000; Neggers et al., 2005) and the PFC. As previous research also found prefrontal areas to be related to attentional modulation (e.g., Csibra et al., 1997), prefrontal responses were extracted in electrode clusters around the left prefrontal electrode FP1 and right prefrontal electrode FP2 between 120 and 180 ms after target onset. Peak latencies were calculated as the latency of the maximum (peak) amplitude within a predefined time window.

The average individually calculated noise threshold for ERP samples (based on the individual subject’s MAD threshold) was 13.6 μV (SD = 6.80 μV) for saccade trials and 13.6 μV (SD = 5.29 μV) for manual response trials, *t*_(23)_ = −0.45, *p* = 0.965, and the individual range threshold in amplitude was on average 53.9 μV (SD = 26.2 μV) for saccades and 53.1 μV (SD = 18.5 μV) for manual responses, *t*_(23)_ = 0.15, *p* = 0.881. The mean number of trials subjects successfully completed behaviorally was 395 (SD = 9.83) trials per subject for saccades and 387 (SD = 24.7) for manual responses, *t*_(23)_ = 1.67, *p* = 0.109. After exclusion of noisy EEG data, an average of 368 (SD = 15.8) saccade trials and 357 (SD = 29.0) manual response trials per subject remained in the analysis, *t*_(23)_ = 2.11, *p* = 0.046.

## Results

**Figure 3:**
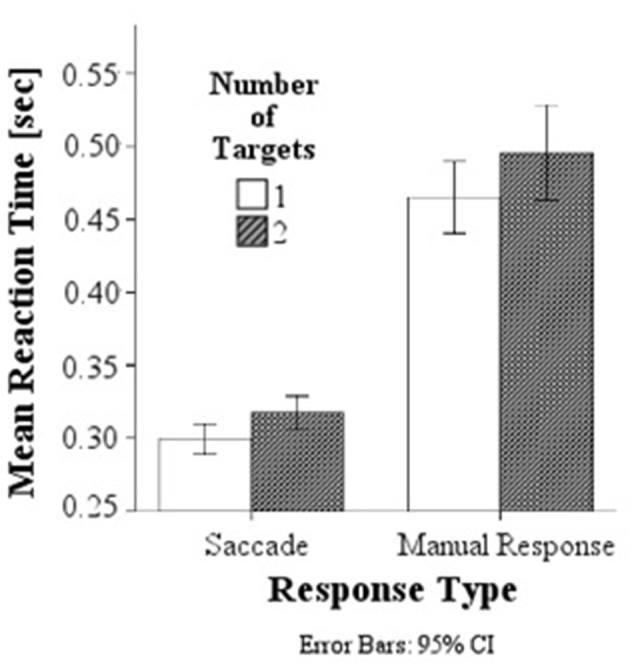
**Mean response latencies for saccades and manual responses.** Responses are slower in the double target condition than in the single target condition for both saccadic and manual responses.

A comparison of behavioral response latencies showed significantly shorter latencies in saccadic conditions (*M* = 0.308 s, SD = 0.038) than in manual response conditions (*M* = 0.480 s, SD = 0.098), *F*_(1,154)_ = 521.61, *p* < 0.001, *d* = 2.317, and as predicted, significantly higher latencies in the double target condition with a target presented simultaneously to the left and right (*M* = 0.406 s, SD = 0.121) than to single targets (either on the left or right; *M* = 0.382 s, SD = 0.104), *F*_(1,154)_ = 10.35, *p* = 0.002, *d* = 0.214, Figure 3. There was no interaction between response modality and number of targets.

**Figure 4:**
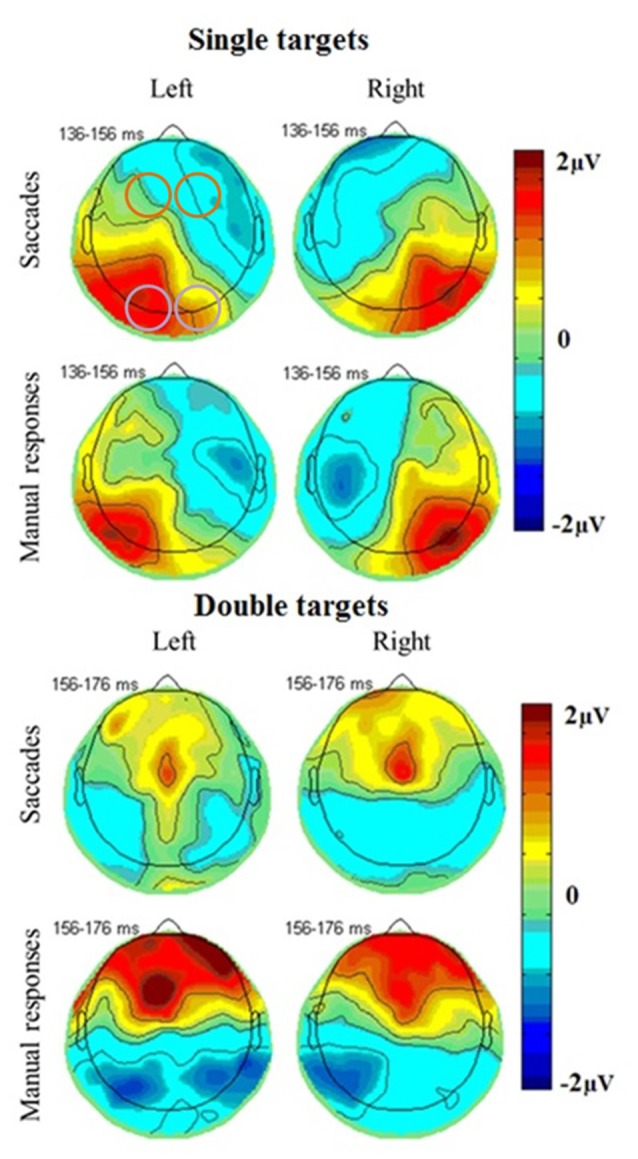
**Topographical plots of EEG responses in single (top) and double (bottom) target conditions averaged across all subjects.** The electrode clusters extracted for analyses are marked in orange (frontal cluster around FC3 [EGI 12, 13, 19, 24, 20, 28, 29] and FC4 [EGI 4, 5, 111, 112, 117, 118, 124]) and purple (occipital cluster O1 [EGI electrodes 65, 66, 70, 71, 69, 74] and O2 [EGI electrodes 90, 84, 76, 83, 82, 89]) in the first plot. A posterior response to single targets on the left (left plots) and right (right plots) side of the screen is more pronounced in the ipsilateral hemisphere for both saccades (top) and manual responses (bottom) and coincides with a fronto-central negativity. Double target trials in which subjects subsequently responded to the right (right plots) or left (left plots) target show a late frontal positivity coinciding with a posterior negativity for both saccades (top) and manual responses (bottom) that is not lateralized with respect to the selected target but slightly more pronounced in the left side of the brain.

The most prominent neural responses in the extracted time window were an occipital positivity that peaked in contralateral areas first, followed by a greater ipsilateral positivity, coinciding with a frontal negativity. In covert attention shift trials and in double target conditions, there was also a frontal positivity peaking towards the end of the extracted time window (Figure 4). ERPs were further analyzed using mixed linear models, including subjects as random effects and response type, number of targets, brain hemisphere and brain side as factors, including also their interactions.

### Posterior Positivity

**Figure 5:**
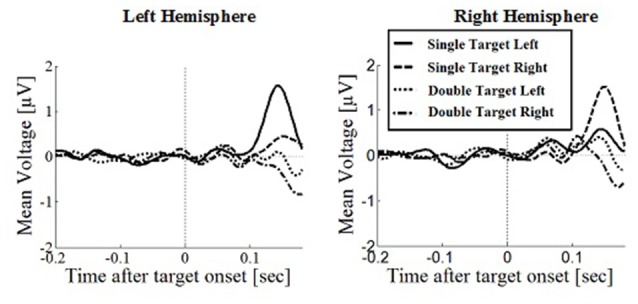
**Wave plot of the occipital response for *saccade conditions* in the left hemisphere (left) and the right hemisphere (right) of the brain**.

**Figure 6:**
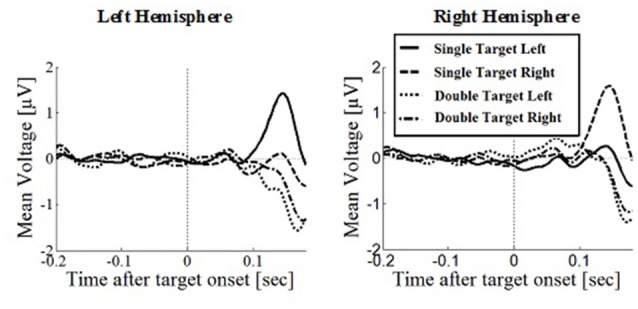
**Wave plot of the occipital response for *manual response conditions* in the left hemisphere (left) and the right hemisphere (right) of the brain**.

The posterior positivity (Figures 5, 6) peaked around 141 ms after target onset (SD = 26.2) with an average amplitude of 1.42 μV (SD = 2.63 μV). Peak amplitude was significantly affected by number of targets, *F*_(1,330)_ = 23.59, *p* < 0.001, *d* = 0.457, with greater amplitudes for single (*M* = 2.01, SD = 2.54) than for double targets (*M* = 0.83, SD = 2.60). No other main effects or interactions were significant. In particular, there was no significant effect of response type on peak amplitude, *F*_(1,330)_ = 0.62, *p* = 0.430. Follow up Bayesian analyses were conducted using the lmBF function of the “BayesFactor” Package (Morey and Rouder, 2015) in Core Team, R (2012) using Cauchy priors based on Liang et al. (2012). Comparing a mixed model including response type with a model excluding response type, suggests that it is 6.7 times more likely that there is no effect of response type than that there is one (*B*_10_ = 0.149 ± 0.94%).

Peak latencies showed a significant effect of number of targets, *F*_(1,330)_ = 20.16, *p* < 0.001, *d* = 0.348, with significantly longer latencies for single targets (*M* = 146, SD = 26.1) than for double targets (*M* = 136, SD = 29.5). There was an effect of response type, *F*_(1,330)_ = 19.08, *p* < 0.001, *d* = 0.364, with significantly longer latencies in saccade conditions (*M* = 145, SD = 26.1) than in manual conditions (*M* = 136, SD = 25.5), contradicting the hypothesis that occipital responses are the same in overt and covert shift conditions. No other main effects or interactions were significant.

### Frontal Negativity

A frontal negativity peaked around 136 ms after target onset (SD = 27.5), measured as the latency of the minimum potential in this area, with an average amplitude of −1.18 μV (SD = 5.40). Peak amplitudes showed no significant main effects or interactions. Its peak latency was significantly affected by the number of targets, *F*_(1,330)_ = 12.04, *p* < 0.001, *d* = 0.290, with longer latencies for single (*M* = 140, SD = 24.5) than for double targets (*M* = 132, SD = 29.7), and response type, *F*_(1,330)_ = 22.64, *p* < 0.001, *d* = 0.405, with longer latencies for saccades (*M* = 142, SD = 27.7) than for manual responses (*M* = 130, SD = 26.2). This reflected the same pattern also observed for the posterior positivity and confirming that there was a difference in neural responses between covert and overt attention shifts. In addition, there was an interaction of target number and response type, *F*_(1,330)_ = 8.30, *p* = 0.004, with greater latency differences between response types for double than for single targets.

### Prefrontal Positivity

**Figure 7:**
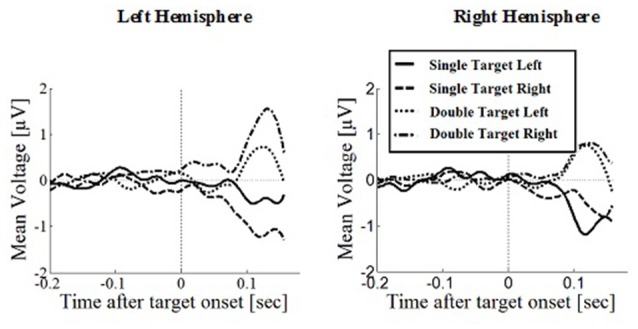
**Wave plot of the prefrontal response for *saccade conditions* in the left hemisphere (left) and the right hemisphere (right) of the brain**.

**Figure 8:**
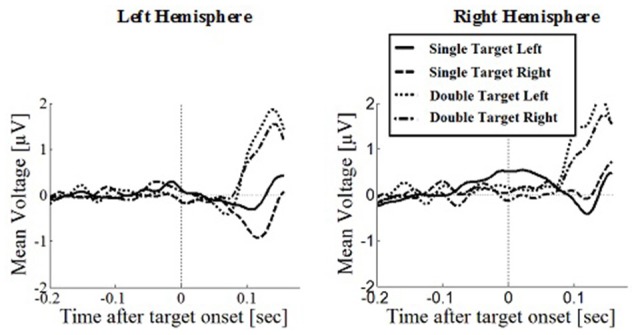
**Wave plot of the prefrontal response for *manual response conditions* in the left hemisphere (left) and the right hemisphere (right) of the brain**.

**Figure 9:**
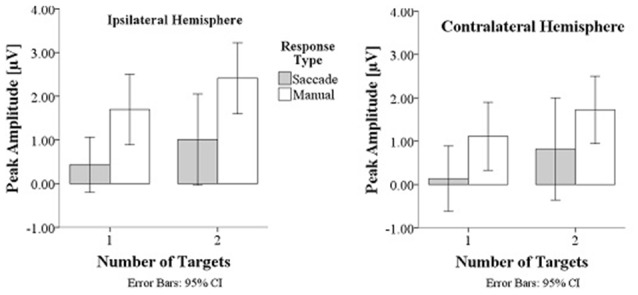
**Peak amplitudes are greater in manual response conditions than in saccade conditions and greater for double than for single targets**.

Prefrontal peak amplitudes (Figures 7, 8), measured as the maximum potential measured in the prefrontal electrode cluster, showed a significant effect of number of targets, *F*_(1,330)_ = 5.74, *p* = 0.017, with smaller amplitudes to single (*M* = 0.842, SD = 2.56) than to double targets (*M* = 1.49, SD = 3.30), of response type, *F*_(1,330)_ = 17.71, *p* < 0.001, with smaller amplitudes for saccades (*M* = 0.597, SD = 3.12) than for manual responses (*M* = 1.74, SD = 2.69). There was an interaction effect of target number, brain hemisphere and response type, *F*_(1,330)_ = 3.91, *p* = 0.049, Figure 9. No other main effects or interactions were significant.

Peak latency showed a significant main effect of response type, *F*_(1,330)_ = 20.31, *p* < 0.001, with shorter latencies in saccade trials (*M* = 146, SD = 23.4) than in manual response trials (*M* = 154, SD = 23.1), which is in line with the hypothesis that there are differences between covert and overt attention shift conditions. No other main effects or interactions were significant.

As described in the “Materials and Methods” Section, the manual task was always run first to avoid transfer of learned saccade responses to the manual task where fixation was required. This raises the question of whether any of the differences between manual and saccade results can be ascribed to order effects. Mixed linear models showed that response times did reduce significantly with trial number within each condition of the experiment, suggesting a learning effect, *F*_(1,399)_ = 3.37, *p* < 0.001. This reduction occurred in both the manual response block, *F*_(1,399)_ = 2.90, *p* < 0.001, and the saccadic response block, *F*_(1,399)_ = 1.82, *p* < 0.001, suggesting that it was not limited to either block. However, there was no interaction of trial number with number of targets, *F*_(1,399)_ = 1.02, *p* = 0.429, or with the side of the target responded to, *F*_(1,399)_ = 1.00, *p* = 0.491, suggesting that the order effect is non-specific and cannot explain the differences in the pattern of responses between manual and saccadic conditions.

## Discussion

### Behavioral Response Latencies

The shorter response latencies in single compared to double target conditions suggest that additional processing effort is required for the decision process as to whether to attend and shift gaze to the target on the left or right, when the left and right targets are presented simultaneously.

### Occipital Responses

The posterior positivity is similar to occipital responses observed in previous studies of covert attention shifts (e.g., Harter et al., 1982; Rugg et al., 1987; Heinze et al., 1990; Luck et al., 1990; Mangun, 1995). The amplitude was higher for single than double targets in both hemispheres, suggesting competitive interactions similar to those found in electrophysiological single cell recordings in non-human primates (Moran and Desimone, 1985) and in human steady-state visually evoked potential studies (Keitel et al., 2013). The posterior response was lateralized only for single targets, with greater ipsilateral amplitudes, but not for double targets, confirming this response at least partially depends on the visual input, as suggested by previous research, rather than attention processing. Response amplitudes did not significantly differ between covert and overt shift conditions. Therefore, the suggestion that saccade planning can enhance brain activity related to visual processing (Saber et al., 2015) was not confirmed in this study. Enhancement due to planning a saccade may not be significantly different from that due to planning a lateralized manual response.

In stimulation studies, occipital cortex has previously been linked to saccade generation, as electrical stimulation of the lower V1 layers facilitates eye-movements (Schiller and Tehovnik, 2005). The current study shows greater activation in electrode sites that correspond to occipital areas for single targets, which might result in the faster saccade latencies seen in this condition. As the early visual cortex projects to the SC (Schiller and Tehovnik, 2005), stimulation of which elicits eye movements (Schiller and Stryker, 1972), occipital responses might directly influence SC activation. SC activation needs to reach a specific threshold for a saccade to be executed (Neggers et al., 2005). Hence, considering previous literature on brain mechanisms of saccade initiation, the higher activation in occipital areas for single than for double targets might result in the threshold for saccade execution being reached earlier in SC, leading to shorter saccadic latencies for single than for double targets. However, it must be noted that the EEG methods used here do not give a direct indication of the source of the responses. It is possible that bilateral stimuli may create partially opposed dipole sources.

The response peaked earlier for double targets than for single targets and it peaked earlier for covert than for overt shifts. This is reminiscent of the shorter EEG latencies reported by Miniussi et al. (1998) in the “redundant target effect”. However, Miniussi et al found that multiple targets led to shorter behavioral latencies, while our result is the opposite. This presumably reflects the task difference in that our participants had to make an additional choice as to whether to make their response to the left or right target, in the double-target trials.

Covert shifts and double target conditions are related to two different variables (response type and visual input). However, both involve an inhibition of an eye movement, either to both sides due to the instructions to covertly shift attention (i.e., a general inhibition of all eye movements), or towards one of the stimuli for the double target condition. In manual response conditions, a clear posterior negativity was visible towards the end of the extracted window. One explanation for the earlier peaking posterior positivity in double target conditions and in manual response conditions may be that their inhibition of eye-movement coincides with a posterior negativity that follows the positivity. Due to the overlap of both responses, the positivity peaks earlier and only lasts for a shorter time. In line with the idea of visual cortical areas activating SC to initiate saccades, it is possible that this inhibitory response may be involved in inactivating SC to prevent eye movements. However, other methods such as studies using MRI would be required to directly investigate the involvement of the SC. Double target conditions also showed smaller peak amplitudes, which is in line with this explanation, as the overlap with the subsequent negativity might also cause this decreased amplitude.

An alternative explanation would be that different mechanisms control the saccade initiation in single and double target conditions. In single target conditions the bottom-up target response (i.e., the response elicited in the visual cortex) is the most reliable source for information to make an eye movement in the correct direction. Hence the visual response drives the fixation shift, indicated by high peak amplitudes in occipital areas. However, in double target conditions subjects can decide freely and therefore use top-down attentional mechanisms to decide which target to shift to and inhibit a saccade to the other target. Therefore, the visual response is smaller, while other responses may play a greater role for initiation of fixation shifts.

### Differences Between Overt and Covert Attention Shifts

Similar occipital responses were found for overt and covert attention shifts. However, the posterior positivity was more temporally extended for overt than for covert attention shifts, possibly due to an earlier posterior negativity in covert attention shift trials which overlapped in time with the positive occipital response. A frontal negativity peaked earlier for covert attention shifts and may be involved in initiating this posterior negativity. Both negativities may reflect processes involved in the inhibition of eye movements. As visual areas can activate SC to initiate eye movements, the inhibitory responses may need to peak earlier to inhibit visual areas from reaching the required threshold. There were no striking differences in early response components between overt and covert shifts in fronto-central areas, as might have been expected due to saccade planning. This suggests that these preparatory processes may be more linked in time to the saccade onset than to the target offset and so not be visible when the extracted potentials were averaged time-locked to the target onset. However, manual response conditions and double target conditions showed a later prefrontal positivity occurring towards the end of the extracted time window. Differences in manual response conditions may be related to manual response planning. However, a major difference is the inhibition of saccades which would occur in normal behaviors, either towards one of two stimuli (double target condition) or overall in the covert attention shifts of the manual response condition. Thus the frontal positivity seen in these conditions may be attributed to the inhibition of saccades.

Most studies of covert vs. overt attention involve instructing the participant to attend to a particular region of the field via a centrally presented cue (e.g., Heinze et al., 1990, 1994; Luck et al., 1990; Mangun et al., 1997; Kelley et al., 2008; Tamber-Rosenau et al., 2011), and so can be considered as an endogenous direction of attention. In contrast, our experiment provided an exogenous trigger for attention, by the appearance of a target in a peripheral field location. Thus it is possible that a different pattern of activation would be seen in the covert direction of attention by an endogenous cue. Nevertheless, the two situations have in common the key factor that, without specific instructions, a participant would normally direct their gaze towards the target location, whether as a preparatory act following a cue, or as a direct response to the target appearance. Thus in both the cued paradigm, and in our “covert” manual response condition, inhibition of a saccade is required. The results of EEG experiments in covert endogenous attention are likely to include the effects of brain mechanisms involved in this saccade inhibition, which cannot easily be separated from those involve in directing attention. These studies need to be complemented by studies of more natural overt attention shifts, before they can lead to firm conclusions about attention mechanisms.

Alternatively, other differences between manual and saccadic tasks may account for part of this difference. Stimuli were kept constant between conditions and the responses were held as simple as possible, to avoid other factors affecting the results. The main reasons for keeping responses simple was to create a bridge between the Fixation Shift Paradigm that is used in non-verbal populations and previous attention paradigms requiring verbal instructions. In populations that can be verbally instructed, it would be possible to use an additional compound manual discrimination task, where participants are required to shift either covert or overt attention to a peripheral target, and then (in both conditions) make a manual discrimination response based on a target feature. In the present study, we cannot exclude the possibility that the planning of manual responses is responsible for some part of the difference between responses in the two conditions.

It is important to note that many previous studies investigating covert attention shifts used an endogenous cue (e.g., Harter et al., 1982; Posner et al., 1984; Rugg et al., 1987; Heinze et al., 1990, 1994; Luck et al., 1990; Mangun, 1995; Müller and Hillyard, 2000), while overt attention shifts were investigated using exogenous cuing paradigms (Csibra et al., 1997, 1998). The current study bridges both findings by investigating both covert and overt shifts using exogenous cuing, therefore filling the gap of exogenous covert cueing studies and allowing a direct comparison of attention shifts with and without eye movements.

Several studies (e.g., Walter et al., 2012; Fimm et al., 2015) have argued that distinct neural mechanisms underlie covert and overt shifts of attention. Our results are more consistent with evidence that the underlying processes are strongly overlapping (de Haan et al., 2008; Krauzlis et al., 2013). The relatively minor differences that we identify are consistent with the idea that covert shifts of attention require additional activity in circuits necessary to actively inhibit a saccadic shift to the target.

### Suitability of the Method

The current study succeeded in measuring brain responses involved in both covert and overt shifts of attention by combining and co-registering eye tracking and EEG. Functional MRI has been used to investigate covert and overt attention shifts (e.g., Corbetta et al., 1998; Kelley et al., 2008), but its low temporal resolution does not allow eye movements artifacts to be excluded unless subjects are instructed to delay their eye movements (memory guided saccades, e.g., Saber et al., 2015). The temporal resolution of EEG allows selection of data from the period before saccade onset (here up to 180 ms from target onset). Data from the eye-tracker can be used to exclude signals that are recorded during eye movements, without the need for subjects to artificially delay or inhibit their saccades. This leads to more natural saccades in response to stimuli and allows a detailed investigation of the time course of responses in different brain areas. However, one drawback of the current methodology is that it only allows investigations of early ERP components which are not affected by artifacts due to eye-movements and the change in visual input related to them. Thus for example the P300 which has been extensively investigated as a signature of attention (e.g., Donchin et al., 1984; Nieuwenhuis et al., 2011) was outside the scope of our approach.

This study was based on the Fixation Shift Paradigm, which can be used as a clinical diagnostic tool in non-verbal populations and infants (e.g., for a review see Atkinson and Braddick, 2012). The methodology developed in this article can therefore potentially be used to investigate neural mechanisms of attention shifts in infants and patients with language impairments (Kulke et al., 2014).

### Conclusion

In this study a novel methodology combining eye tracking and EEG was used to investigate the difference in neural responses for shifts of attention involving either an overt eye-movement or a manual response without change of fixation. There were some similarities in covert and overt attention shift tasks, particularly in occipital areas, suggesting that these responses may be part of the neural processing underlying the attention shifts necessary for perceptual and cognitive tasks in everyday life. In the covert, compared to overt attention task the main difference involves a frontal positivity that is likely to reflect saccade inhibition, due to the requirement to maintain central fixation and inhibit eye movements to the peripheral target while choosing to make a manual response to either the left or right target. The results suggest caution in interpreting ERPs in covert attention shifts, since these may reflect this inhibitory process rather than isolated processes of spatial selective attention.

## Author Contributions

LVK was involved in the conception and design of the work, the acquisition, analysis and interpretation of data, drafting the work and revising it critically for important intellectual content. JA and OB were involved in the conception and design of the work, interpretation of data and critical revision of the manuscript for intellectual content. All authors gave final approval of the version to be published and agree to be accountable for all aspects of the work.

## Conflict of Interest Statement

The authors declare that the research was conducted in the absence of any commercial or financial relationships that could be construed as a potential conflict of interest.
